# Biomechanical Force Prediction for Lengthening of Small Intestine during Distraction Enterogenesis

**DOI:** 10.3390/bioengineering7040140

**Published:** 2020-11-07

**Authors:** Hadi S. Hosseini, James C. Y. Dunn

**Affiliations:** 1 Department of Surgery, Division of Pediatric Surgery, Stanford University School of Medicine, Stanford, CA 94305, USA; seyyed.hadi.hosseini@gmail.com; 2 Department of Bioengineering, Stanford University School of Medicine, Stanford, CA 94305, USA

**Keywords:** distraction enterogenesis, computational modeling, short bowel syndrome, small intestine biomechanics

## Abstract

Distraction enterogenesis has been extensively studied as a potential treatment for short bowel syndrome, which is the most common form of intestinal failure. Different strategies including parenteral nutrition and surgical lengthening to manage patients with short bowel syndrome are associated with high complication rates. More recently, self-expanding springs have been used to lengthen the small intestine using an intraluminal axial mechanical force, where this biomechanical force stimulates the growth and elongation of the small intestine. Differences in physical characteristics of patients with short bowel syndrome would require a different mechanical force—this is crucial in order to achieve an efficient and safe lengthening outcome. In this study, we aimed to predict the required mechanical force for each potential intestinal size. Based on our previous experimental observations and computational findings, we integrated our experimental measurements of patient biometrics along with mechanical characterization of the soft tissue into our numerical simulations to develop a series of computational models. These computational models can predict the required mechanical force for any potential patient where this can be advantageous in predicting an individual’s tissue response to spring-mediated distraction enterogenesis and can be used toward a safe delivery of the mechanical force.

## 1. Introduction

Short bowel syndrome (SBS) is a devastating malabsorptive condition associated with a lack of functional intestinal surface area of bowel. Due to this lack of absorptive surface area, the remnant intestine cannot adequately absorb nutrients, resulting in the inability of the enteral source of nutrition to sustain growth, development and life [1,2]. Complications from SBS frequently result from medical therapies to support this condition, which include but certainly are not limited to line infections from the need for central venous access and liver disease associated with chronic parental nutrition. Different approaches have been investigated as treatments for SBS, including supportive measures such as parenteral nutrition [3,4], medications to increase intestinal absorptive capacity by slowing transit and invasive surgical lengthening procedures such as serial transverse enteroplasty [5,6]. However, these approaches have relatively high morbidity and mortality rates, and their complications are costly to manage compared to the level of success to achieve enteral autonomy [5,7,8,9]. For example, intestinal transplantation has a graft rejection estimated to affect approximately 50% of patients by five years with a similar rate of mortality [8,9].

Generating novel therapies may obviate the need for the current methods of treatments and their high complication rates in patients with SBS. Mechanical stimulus has been extensively examined as an important factor in stimulating tissue growth and proliferation. Distraction enterogenesis was proposed as a novel method to increase intestinal length and absorptive surface area. Distraction enterogenesis relies on applying a mechanical force to a segment of intestine to promote intestinal lengthening; a variety of methods and device considerations have been investigated to incorporate the external mechanical force to the distracted segment of bowel [10,11,12,13,14,15,16,17,18,19,20,21].

**Figure 1:**
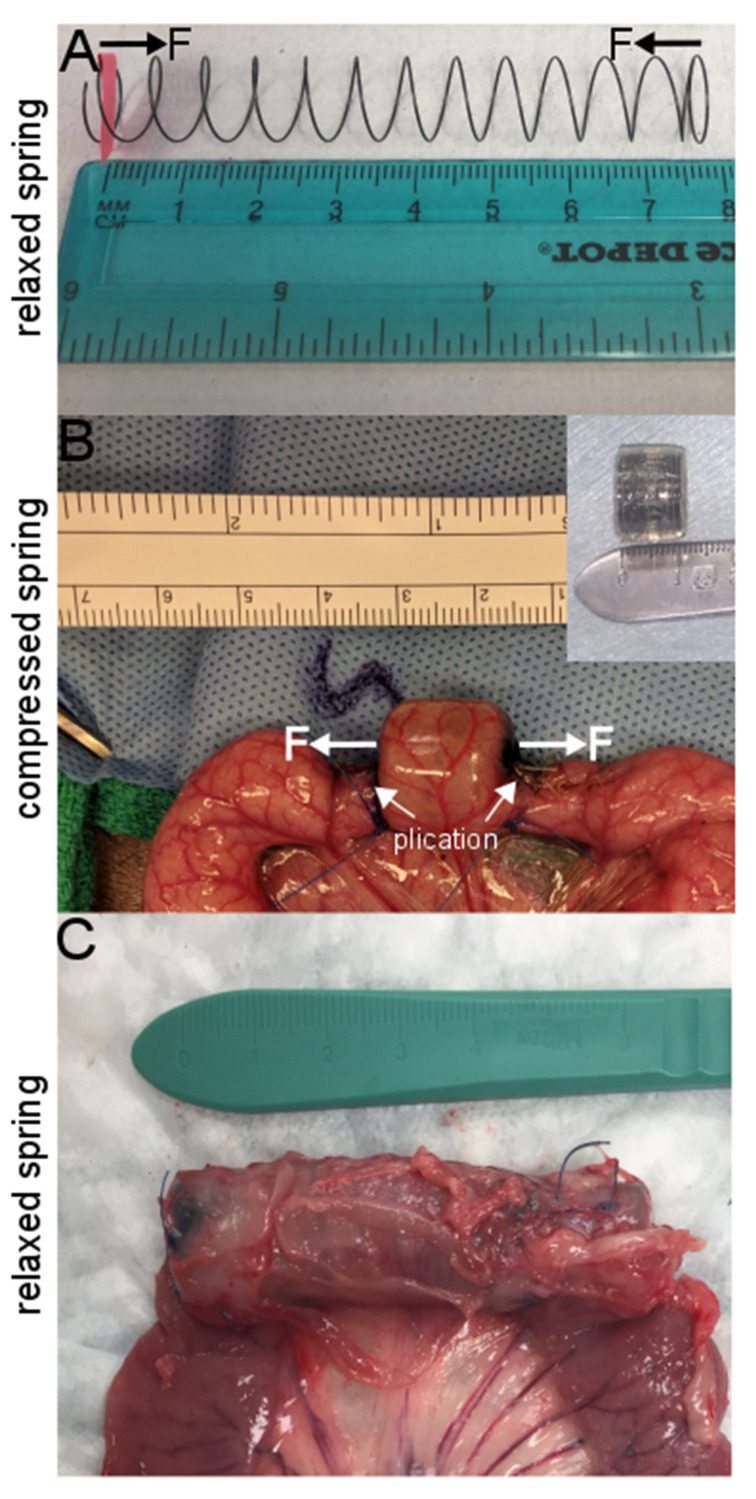
Representative images for distraction enterogenesis experimental setup using intraluminal spring. (**A**) Relaxed spring. (**B**) Intra-operative image demonstrating compressed encapsulated spring within a segment of small intestine held in place with two plication sutures. (**C**) Distracted segment of small intestine with expanded intraluminal spring at time of tissue retrieval.

Previously, we developed a method of distraction enterogenesis using an intraluminal spring (Figure 1) where an axial mechanical force was applied within the lumen of the intestine to lengthen the intestine, and this approach has been performed successfully in several animal models [10,11,12,15,18,19,20,21,22]. Our studies confirmed that mechanical perturbations in the axial direction of the intestinal tract trigger signaling pathways that cause tissue thickening in the radial direction within the distracted segment, as well as adaptive responses in the areas adjacent to the distracted segment [23,24,25].

To deliver a safe and efficient outcome for distraction enterogenesis using the self-expanding intraluminal spring, we need to scale the applied mechanical force based on intestinal size to ensure that the delivered physical force is customizable based on the physical characteristics of the small intestine for each subject. We previously showed the general scalability of our method between different animal models (mouse, rat and pig) [16,23,25,26]. In this study, we further investigated these criteria with a focus on human subjects. First, we collected data from human tissues to better understand the range of changes in geometrical characteristics (thickness and diameter) of the small intestine for human patients. We then performed mechanical characterization of the intestinal tissue, where these findings were used for our computational modeling efforts. We previously developed a computational modeling platform to study and predict the small intestinal tissue response to applied mechanical force [23]. Here, we further extended the computational model specifically for human subjects and developed a series of models for any potential size of human small intestine.

## 2. Materials and Methods

### 2.1. Spring Production and Encapsulation

As previously described [23,24], biocompatible nickel-titanium (nitinol) springs were created where a 0.02 inch gauge nitinol wire (McMaster-Carr, Santa Fe Springs, CA, USA) was wrapped around a 1.3 cm diameter mold, heated to 500 °C for 30 min, rapidly cooled under water. Springs were cut to 7.5 cm in length, and spring constants measured (Figure 1A). Springs were compressed and placed within an absorbable gelatin capsule (Fisher Scientific, Pittsburgh, PA, USA) and then coated with cellulose acetate phthalate (Eastman Chemicals, Fairfield, NJ, USA), which allows for delayed gelatin capsule degradation (Figure 1B).

### 2.2. Animal and Human Sample Preparation and Surgical Procedure

Animal surgeries and care were approved by Stanford Administrative Panel on Laboratory Animal Care (protocol 32278). Four- to six-week-old juvenile female Yucatan pigs (S&S Farms, Ramona, CA, USA) underwent intervention. Animal subjects underwent general anesthesia and were sterilely prepped and draped [25]. A midline laparotomy incision was made, the jejunum identified 50 cm from the Ligament of Treitz. An anti-mesenteric longitudinal incision was made at this point in order to introduce an encapsulated spring. India ink was injected into the submucosa to identify a 1.5 cm long segment to identify the region of compressed spring-loaded capsule (Figure 1). Once the capsule was introduced to this marked segment, the bowel was plicated to 50% of bowel diameter (Figure 1B) to ensure that the expanded spring remained in place: proximally by two 4-0 polypropylene interrupted sutures and distally by four sutures. The enterotomy was then closed primarily, the small intestine returned into the abdomen, the abdomen irrigated, and the incision closed in multiple layers. Animals were provided with liquid diets for the first post-operative week.

### 2.3. Geometrical and Histologic Evaluation

**Figure 2:**
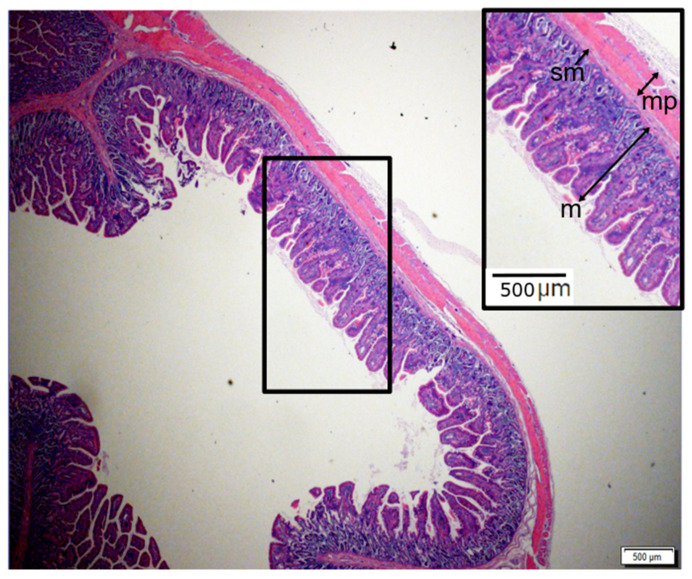
Representative cross-sectional light micrographs of H&E stained jejunum under normal (zero force) conditions. Black arrows indicate major layers of intestinal wall as mucosa (m), submucosa (sm) and muscularis propria (mp).

Pigs were euthanized, and bowel segments were retrieved. Normal segments and segments of intestine containing the springs were removed and evaluated for lengthening as well as for histologic examination. Intestinal segments were placed in 10% buffered formalin (Fisher Scientific, Pittsburgh, PA) overnight. Samples were then cut into cross-sections and imbedded in paraffin. Paraffin blocks were cut into 5-μm sections to create slides that were stained with hematoxylin and eosin (H&E) (Figure 2). The thickness of each layer of intestinal wall was measured at multiple representative locations on each slide and averaged to calculate the mean for each section.

**Figure 3:**
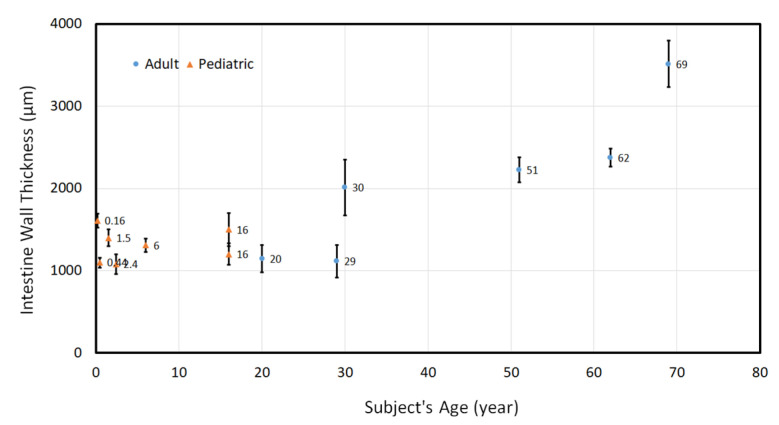
Geometrical metrics measurement of small intestine of a wide range of human subjects. Total number of human subjects was n = 61 for the full age range.

Human small intestines were collected as discarded, de-identified tissue at the time of surgery as approved by the Stanford Institutional Review Board. For human samples, they were rinsed with saline to remove intestinal content and then were placed in phosphate buffered saline (PBS) on ice to keep them fresh. First, they were flattened on a glass slide to perform diameter and thickness measurements (Figure 3) using a fine caliper; then, each sample was prepared either for mechanical characterization or H&E staining. Specimens for mechanical testing continued to be kept in PBS (see Section 2.4) while samples for H&E staining underwent a similar process as pig specimens as described above.

### 2.4. Mechanical Characterization

**Figure 4:**
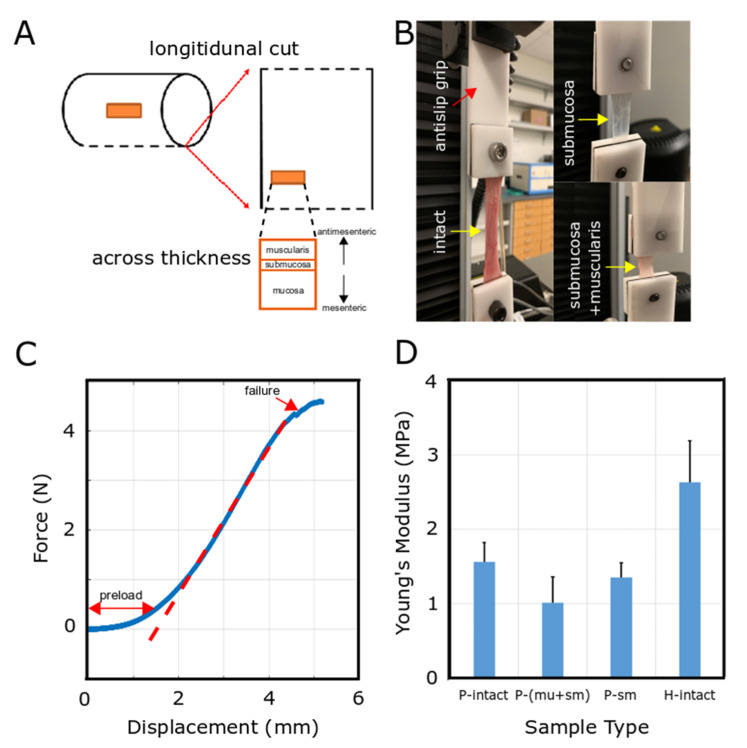
Mechanical characterization of pig and human small intestinal tissue. (**A**) Schematic diagram represents how rectangular specimens were prepared from cylindrical small intestinal tract. (**B**) Tensile test performed using an Instron type 5565 with anti-slip grips. (**C**) Representative force-displacement plot of a specimen. (**D**) Average Young’s modulus for pig and human. Sample numbers are n = 36, 27, 39, 31 for P-intact, P-(mu+sm), P-sm and H-intact, respectively, while subject (pig and human) numbers are n = 7, 5, 6, 7 for P-intact, P-(mu+sm), P-sm and H-intact, respectively.

Immediately after euthanizing the animal, a laparotomy was performed to remove 10–20 cm segment of small intestine (jejunum). Specimen was rinsed with saline to remove intestinal content and the specimen was placed in PBS on ice. Segments of freshly harvested porcine small intestine were stored in PBS prior to uniaxial tensile testing. Cylindrical intestinal tract was cut open (Figure 4A), and longitudinal cuts were made to obtain the elastic modulus of the small intestine in the longitudinal direction. To perform mechanical testing for intact tissue, no further procedure was needed. For mechanical characterization of submucosa and muscularis layers together, the mucosal layer was removed using a glass slide to scrape off the mucosa. In another set of mechanical tests for the submucosal layer, both mucosal and muscularis layers were removed from both sides of submucosa.

Tensile tests were performed using an Instron type 5565, with a 1 kN load cell to measure the elastic modulus. Specimens were clamped using special custom-made anti-slip grips to counteract any slipping (Figure 4B). A pre-load of 2 N was then applied at a displacement of 0 mm to eliminate slack within the sample. After pre-load step, the force and displacement measurements were stored using the controlling software while the mechanical load stretched the specimen due to applying axial mechanical force (Figure 4B,C). Test was stopped once failure was observed in the force-displacement plot (Figure 4C).

### 2.5. Computational Methods

Finite element analysis has been extensively used to study problems with a wide range of biomedical applications in different organs of the body [23,27,28,29,30,31,32,33,34,35,36]. Here, computational models for distracted enterogenesis were developed using the commercial finite-element software ABAQUS (version 2017, SIMULIA, Providence, RI) using implicit solver.

#### 2.5.1. Overview of Models

**Figure 5:**
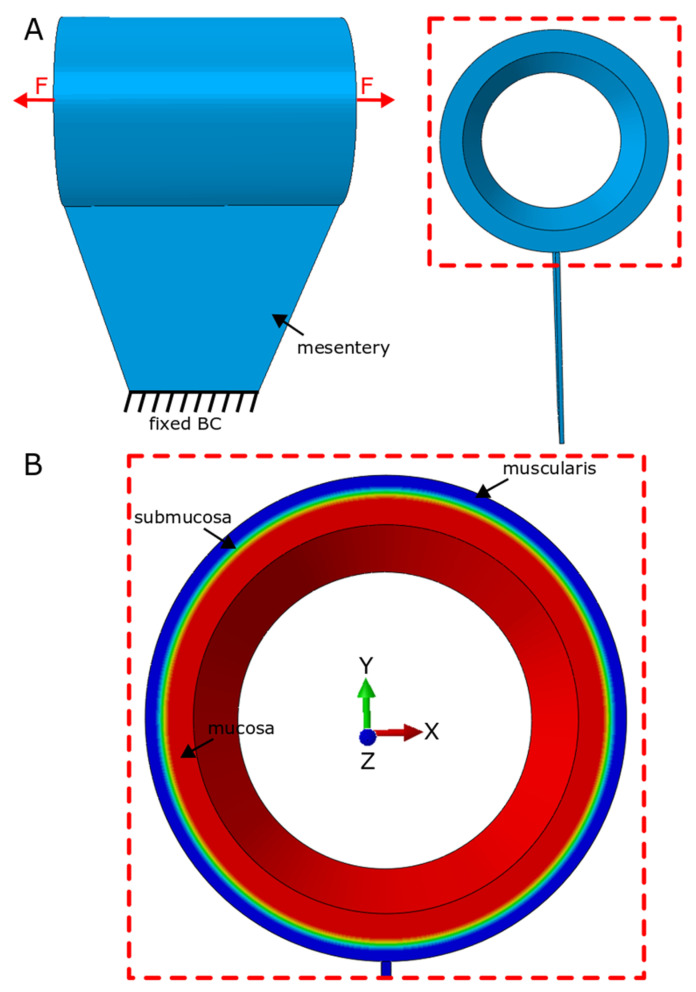
Finite element model for distraction enterogenesis. (**A**,**B**) Frontal and cross-sectional view of the model with mucosa, submucosa and muscularis layers shown in red, yellow/green and red, respectively, in the distracted segment. Model also includes mesentery layer attached to the distracted segment on the mesentery side. Fixed boundary conditions were used for bottom end of mesentery layer. BC and F in (**A**) are abbreviations for boundary condition and force.

Model geometry was based on measurements from histology slides and geometrical measurements of intestinal tissue (Figure 2 and Figure 3). The intestinal tract was considered as a cylindrical hollow, while a thinner layer was tied to the main cylindrical part as the mesentery layer (Figure 5). A series of models were developed to cover the full range for radius and thickness based on our experimental measurements of geometrical characteristics of the human small intestine (Figure 3). The considered radius range was $R_{inner}=0.25–3\;\mathrm{cm}$ while the thickness of intestinal wall was varied t=0.5–3 mm while the initial length of distracted segment was chosen similar to the distracted segment in the experimental setup. For each computational model, the intestinal wall was divided into three main layers: mucosa, submucosa and muscularis propria (Figure 5). Thickness of each layer compared to the total wall thickness is as follows: $\frac{t_{mucosa}}{t_{total}}=0.75,\;\frac{t_{submucosa}}{t_{total}}=0.05,\;\frac{t_{muscularis\;propria}}{t_{total}}=0.2$. These divisions and ratios were approximated from experimental histology images (Figure 2).

Appropriate boundary conditions were enforced for the internal surface of the hollow cylinder of the distracted segment and the bottom part for the mesentery layer throughout the simulation (Figure 5A). In the computational models, the internal surface of the cylinder was chosen to have only displacement freedom (movement in X, Y and Z directions) with no freedom to rotate; this assumption was supported by our experimental observation. During the secondary animal surgery to remove the springs as well as to evaluate lengthening of distracted segment, we observed that the distracted segment was still relatively straight with no sign of tissue rotation (Figure 1), which provides evidence for our assumption of the boundary condition of the internal surface of cylindrical hollow (Figure 1). This is probably due to the close physical contact of the spring with the internal surface of the intestinal hallow. Since the base of the mesentery has very limited movement in vivo, this end of the mesentery layer was fixed throughout the simulation (Figure 5A).

Each model starts with applying mechanical force at both ends, similar to what occurs in the experimental setup. This mechanical force is due to when the compressed spring starts to be relaxed and generates the mechanical stimulus, where it distracts and thus stretches the tissue to lengthen it over time. As the spring lengthens, it stretches the distracted tissue, therefore generating elastic tissue deformation. In addition to this elastic deformation, the mechanical distraction triggers the tissue proliferation component which we previously investigated [23]. The focus of this study, however, is to predict the required mechanical force that generates double elastic lengthening for each model (with different radius or intestinal wall thickness).

Hexagonal elements (C3D8) were used for all parts of the distraction enterogenesis model for all models. For each model, testing with finer meshes confirmed that the chosen mesh size was sufficiently accurate for the present purposes.

#### 2.5.2. Theory

Previous studies have shown great success of solid mechanics theory in studying large deformation of soft tissue involving tissue growth [23,30,31,32]. Distraction enterogenesis processes were simulated using a continuum mechanics theory for large deformation [23,37]. In brief, the theory approximates the soft intestinal tissues as pseudo elastic with negligible viscous effects [38]. Total deformation gradient tensor F, which maps material points from the initial configuration to the deformed configuration at a later time, is decomposed as:

(1) $$F=F^{*}\cdot G$$

where G and $F^{*}$ are the growth tensor (in this study, G =1) and elastic deformation gradient tensor, respectively. As the tissue deforms, F maps the particles between deformed and unreformed configurations.

Because of the cylindrical shape of intestinal tract, in the initial configuration, separate cylindrical coordinate systems (R, θ,Z) are considered for distracted and plication segments. Relative to these coordinates, the growth tensor is taken in the orthotropic form

(2) $$F=F_{R}\;e_{R}e_{R}+F_{\theta }\;e_{\theta }e_{\theta }+F_{Z}\;e_{Z}e_{Z}$$

where the $e_{I}$ are unit base vectors. With the tissues assumed to be slightly compressible, based on the continuum mechanics theory, the constitutive relation has the form [39]

(3) $$\sigma =\frac{1}{J}F\cdot \frac{\partial W}{\partial E}\cdot F^{T}$$

where σ represents the Cauchy stress tensor from the strain-energy density function W(E). In Equation (3), J=detF is the elastic volume ratio and $E=\frac{\left(F^{T}\cdot F-I\right)}{2}$ is the Lagrangian elastic strain tensor, I is an identity tensor and T indicates the transpose. A neo-Hookean strain-energy density function is chosen in the form

(4) $$W=C*\left({\;\bar{I}}_{1}-3\right)+\frac{1}{D}\left[\frac{1}{2}\left(J^{2}-1\right)-\ln J\right]$$

where C is directly related to shear modulus of the intestinal tract tissue and D represents the volumetric compliance, and ${\;\bar{I}}_{1}=J^{2/3}tr\left(I+2E\right)$ is a modified strain invariant. Model constants in Equation (4) were calculated using obtained Young’s modulus and Poisson’s ratio as follows:

(5) $$C=\frac{G}{2}=\frac{E}{4*\left(1+\vartheta \right)}$$

(6) $$D=\frac{2}{K}=\frac{6*\left(1-2\vartheta \right)}{E}$$

where G, K, E and ϑ are shear modulus, bulk modulus, Young’s modulus and Poisson’s ratio, respectively. Young’s modulus (E) was obtained from mechanical characterization results while ϑ was obtained from the assumption that soft tissue behaves in a nearly incompressible manner (ϑ=0.49); then, using E and ϑ, constants C and D were calculated for each layer of intestinal wall.

## 3. Results

### 3.1. Geometrical Characteristics of Human Small Intestinal Tract

Small intestinal samples were collected from discarded human tissues with age range from 2 months to 69 years. Intestinal wall thickness between these subjects varied in the range of ≈1–4 mm (Figure 3). The content of the intestine was washed out, and the cylindrical tract was flattened on a glass slide to measure the distance between the mesentery and anti-mesentery sides (d), which is half of the circumference of the intestine, or $d=\frac{Circumference\;of\;circular\;cross\;section}{2}=\frac{2\pi R}{2}=\pi R$, thus $R=\frac{d}{\pi }$. This process was repeated for all of the intestinal samples, and the range of the radius was ≈1–2 cm.

Wall thickness generally increased with age, consistent with the growth of small intestine as individuals develop. A similar increasing pattern was observed for the radius of intestine.

### 3.2. Mechanical Characterization of Small Intestinal Tissue

Collected data from mechanical testing on the intestinal tissue of pig and human using an Instron type 5565 with a 1 kN load cell were used to calculate the mechanical properties of the tissue. First, the preload and failure parts of the force-displacement plot (Figure 4C) were removed to determine the Young’s modulus using the slope of fitted lines. The same post-processing calculations were repeated to determine the Young’s modulus of intact or separated layers of intestinal tissues.

Averaged Young’s modulus of intact intestine on pig samples was 1.56 MPa, which was higher than that of both submucosa and “submucosa + muscularis propria” layers, where “submucosa + muscularis propria” layers had an average of 1.01 MPa and the average Young’s modulus for the submucosal layer was 1.35 MPa (Figure 4D). The submucosal layer contains the majority of the extracellular matrix, whereas the muscularis propria layer is formed of smooth muscle fibers.

Human intestinal tissue showed significantly higher Young’s modulus with an average of 2.63 MPa as compared to porcine tissue. All of the mechanically tested human tissues were from pediatric subjects. Moreover, due to the limited source of human intestinal samples, mechanical testing was performed only on intact tissue, while for pig samples, mechanical characterization was completed for both intact and separated layers (Figure 4D). The ratio of Young’s modulus between different layers of porcine tissue was employed for simulation purposes.

### 3.3. 3D Computational Model for Distraction Enterogenesis

A series of 3D computational models with different thicknesses for intestinal wall and radius were created to predict the required mechanical perturbation to achieve double lengthening of the small intestine during distraction enterogenesis. For each model, forces were applied at both ends, and the calculations were stopped when the desired double lengthening was achieved.

**Figure 6:**
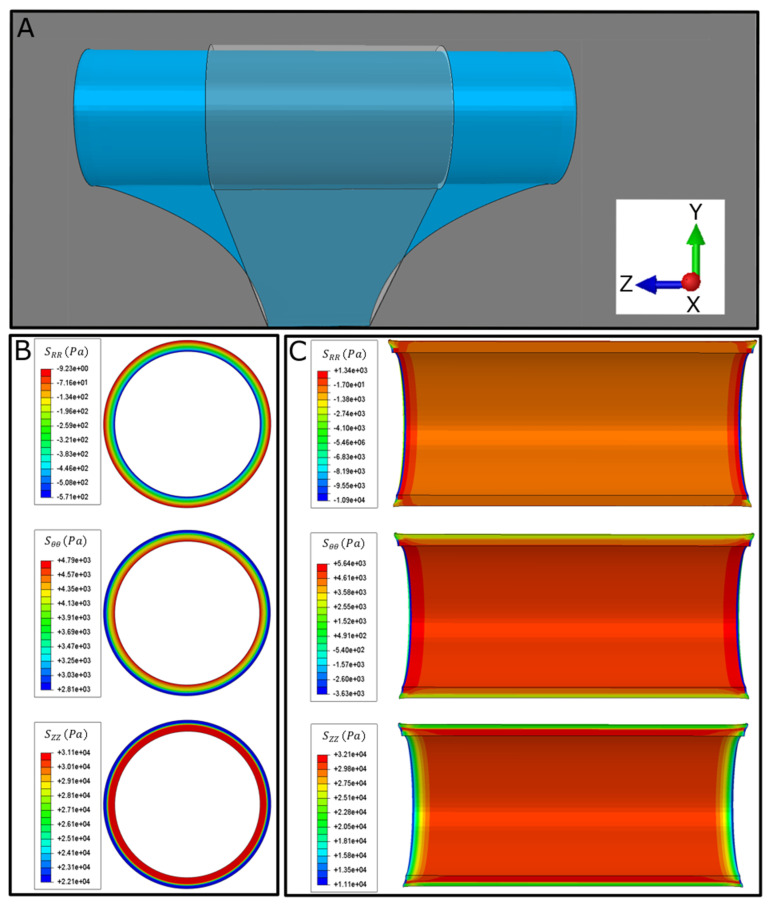
Computational model results after lengthening of distracted segment due to applied mechanical forces at both ends. (**A**) Representative frontal view of model. (**B**,**C**) Stress representation of computational model in R, θ, Z directions.

**Figure 7:**
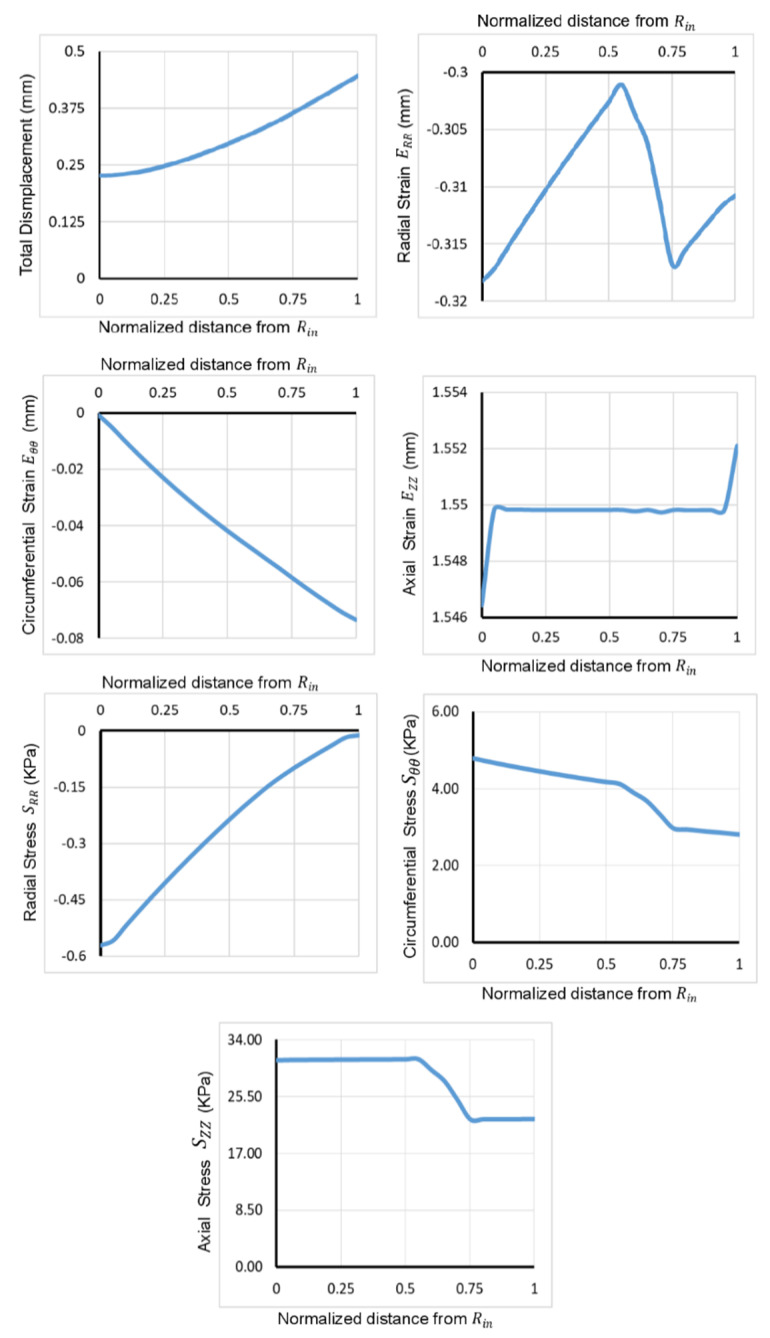
Dispacement, strain and stress in different directions across the thickness of intestinal wall calculated from computataional model results.

In each model, constants C and D for each layer were calculated using the results of mechanical testing. As the force is applied in the axial direction, the tissue initially shrinks in the radial direction and becomes thinner due to the negative stress experienced by tissues in the radial direction and positive stress in the axial and circumferential directions (Figure 6B and Figure 7). An increasing stress pattern was observed for $S_{\theta \theta }$ and $S_{ZZ}$ from the inner to the outer intestinal wall. This stress pattern was decreasing for the radial stress $S_{RR}$ (Figure 6B and Figure 7). No significant change in stress was observed axially (end to end) except for the ends where the mechanical forces were applied (Figure 6C and Figure 7).

Total tissue displacement increased from the inner to the outer intestinal wall. Strain components had different behaviors: $E_{RR}$ had negative value for all values across the thickness and decreased in the mucosa and muscularis propria but increased in the submucosa. Circumferential strain,$\;E_{\theta \theta }$, was negative and decreased across the thickness, whereas axial strain, $E_{ZZ}$, was positive and increased across the thickness (Figure 7).

**Figure 8:**
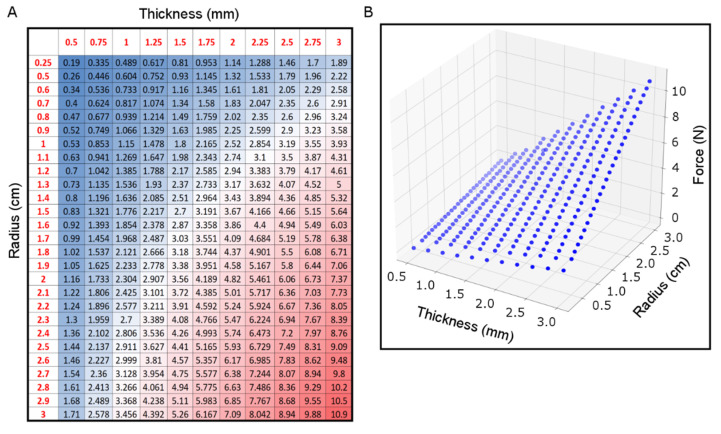
Predicted required mechanical force for different geometrical metrics (radius and thickness) of small intestine for human subjects. (**A**) Heat map and (**B**) Regular 3D plot.

Having an understanding of the required force for each patient is needed to achieve the desired efficiency without applying excessive mechanical force that may damage the intestinal tissue. Computational models were developed for a combination of wall thickness and intestinal radius to predict the magnitude of the mechanical forces needed to double the intestinal length. The range of thickness and radius in the developed models was 0.5–3 mm and 0.25–3 cm, respectively. The predicted forces ranged from 0.19 N for $\left(t=0.5\;\mathrm{mm},\;R_{in}=0.25\;\mathrm{cm}\right)$ to 10.9 N for $\left(t=3\;\mathrm{mm},\;R_{in}=3\;\mathrm{cm}\right)$ (Figure 8). The magnitude of the predicted required force increased with respect to increasing thickness and radius.

## 4. Discussion

Distraction enterogenesis is a recent technique that has been used to lengthen the small intestine in multiple animal models [11,12,13,14,15,16,17,18,19]. Given our prior focus on the effectiveness and practicality of distraction enterogenesis for the intestine, we have expanded our analysis to predict the required mechanical force to achieve double elastic lengthening for any potential size of small intestine for human subjects. A significant variation was observed for small intestine geometrical characteristics such as thickness and diameter. The thickness of each layer of the intestinal wall was measured, where approximately 75% of the wall was the mucosa while the submucosa and muscularis propria comprised 5% and 20% of the total thickness, respectively.

The experimental results on mechanical characterization of intestinal tissue suggest that not only do the mechanical properties of human intestinal tissues differ from those of animals but also that different layers of the intestinal wall have different mechanical properties due to the differences in their biological content. Human intestinal tissue was observed to have significantly stiffer tissue compared to porcine tissues, and the ratio between Young’s modulus of different layers of intestinal wall from these mechanical characterizations were used in the computational models.

A series of computational models to cover the full range of geometrical characteristics variation of the human small intestine were developed here. These models were used to simulate the biomechanical response of the intestinal tissue to the applied force. Due the differential mechanical properties of the layers, the stress and strain of these layers were different accordingly. These computational models were used to estimate the required mechanical force for distraction enterogenesis for each potential small intestinal size. The required forces varied from the smallest and thinnest to the largest and thickest intestine, where this force has an approximately linear relationship with respect to the thickness and diameter of the small intestine.

This is the first study of distraction enterogenesis that utilizes a computational modeling platform refined by experimental observations to predict the required force to build the medical devices—in this case, the self-expanding intraluminal spring. These predicted forces can be used to calculate the spring constant based on Hooke’s law for the design of the geometrical spring characteristics. Modeling becomes critically important for predicting clinical responses including elastic deformation (present study) and tissue proliferation [23]. The model can be scaled based on patient size, intestinal diameter and thickness and can incorporate spring characteristics for more accurate predictions. This proposed use depends on the concept of scalability and assumes that one can rescale the required force for intestinal tracts of different sizes. We previously investigated this scalability assumption, where our results support this based on experimental and computational studies on different animal models [16,23,24]. This computational modeling platform could reduce the risk and uncertainty in surgery by defining optimal spring characteristics (e.g., spring diameter, number of turns, thickness of spring wire, etc.) based on individual patient metrics. This platform also can be used to help surgeons to investigate different surgery conditions such as multiple springs in series, implantation of springs with adaptive features, different loading scenarios that will give further insights into the clinical application of distraction enterogenesis.

Future studies are required to further investigate and improve the model predictions. The geometry of our current model is relatively simple, although it includes the essential features of the experiment. For instance, the distracted segment in our model was considered straight and does not account for the curvature of the intestinal tract. Although the intestinal tract has smooth curvature, we expect this curvature to change the stress experienced by tissue slightly, particularly at both ends where the intestine is plicated with sutures. Therefore, a short distracted segment is not completely straight and further improvement of the model can take this into consideration. Another feature that needs further investigation is the anisotropic behavior of intestinal wall layers [40,41], where the muscularis propria layer is formed of two muscle layers with muscle fibers in different directions. Although several studies have tried to model the anisotropic behavior of the intestinal tract, different theoretical models were offered for strain energy density function for different animal models [40,42,43,44]. These suggested models are for normal conditions, not for cases with perturbations, as is the case in intestine undergoing distraction enterogenesis. Considerations for fiber directions can also be further investigated in future work and added to the computational models. Incorporating additional features into the model will improve its predictive power.

## 5. Conclusions

As a biomedical solution for SBS, an intraluminal spring force lengthens intestinal segments. Knowing the magnitude of this mechanical force is crucial to achieve successful lengthening while minimizing tissue damage. Properties of human intestinal tissue were mechanically characterized as bulk and as separate layers of the intestinal wall. Furthermore, geometrical metrics of a wide range of patients were identified. These experimental measurements were used to develop a series of computational models to cover the whole range of small intestine sizes for human subjects. These models were used to predict this required force to form a reference table that can be used for the intraluminal spring customization for each patient. These findings will stimulate further research on this topic and can ultimately be applied to a safer and more efficient surgical procedure for spring implementation in patients with SBS.

## Dual Publication Statement

We confirm that this work is original and has not been published elsewhere, nor is it currently under consideration for publication elsewhere.
